# Editorial: Multipurpose prevention technologies: call for innovative strategies to address critical priorities and gaps

**DOI:** 10.3389/frph.2024.1417974

**Published:** 2024-05-17

**Authors:** Clara Soh, Ariane van der Straten, Anke Hemmerling, Jim A. Turpin, Bethany Young Holt

**Affiliations:** ^1^ CAMI Health, Initiative for MPTs, Public Health Institute, Oakland, CA, United States; ^2^ ASTRA Consulting and CAPS, Department of Medicine, University of California San Francisco, San Francisco, CA, United States; ^3^ Department of Obstetrics, Gynecology and Reproductive Sciences, University of California, San Francisco, CA, United States; ^4^ TurHow Consulting Group, LLC, Frederick, MD, United States

**Keywords:** MPT, MPTs, multipurpose prevention technologies, HIV prevention, contraception, STI prevention and control, HIV

**Editorial on the Research Topic** Multipurpose prevention technologies: call for innovative strategies to address critical priorities and gaps

When developing new drugs and healthcare interventions, we often focus on the technical challenges. Development of new therapeutics and preventives is difficult and even developing products based on existing technologies poses a challenge. Beyond the scientific hurdles, there are numerous other challenges including fluctuating levels of funding and general underinvestment in certain conditions, socio-behavioral aspects that can affect uptake and product acceptability, and a challenging regulatory, manufacturing and reimbursement environment (1). For products such as multipurpose prevention technologies (MPTs)—which aim to simultaneously prevent HIV, other sexually transmitted infections (STIs), and/or unintended pregnancies—these challenges can be even greater as they combine multiple active pharmaceutical ingredients (API) or drugs into a single product and face complex and sometimes uncharted development pathways.

Building on research on contraception and prevention of HIV and other STIs, the MPT field was launched in 2009 to address these intrinsically linked risks (2–4). As the field matured, field-wide partners refined priority action areas and expanded our focus to encompass the many factors needed to ensure widespread and timely uptake of MPTs when they become available (Table 1) (5). Improved outcomes and equitable access can only be achieved if products reach the hands of end users and these action areas reflect this understanding of the entire product lifecycle. The papers in this special issue are organized around five priority areas; they provide updates, lessons learned, and discussion around what the MPT field can do in order to continue advancing product development and distribution in an efficient and equitable manner.

**Table 1:** Priority areas to advance MPT development and corresponding articles.

1. ***Stimulate a productive ecosystem of MPT research and development (R&D)*** - End-to-end approach to ensuring equitable access to multipurpose prevention technologies in low- and middle-income countries Cameron et al. - Key programmatic and policy considerations for introducing multipurpose prevention (MPT) methods: Reflections from healthcare providers and key stakeholders in South Africa Kutywayo et al. - Innovations in monoclonal antibody-based multipurpose prevention technology (MPT) for the prevention of sexually transmitted infections and unintended pregnancy Dohadwala et al. 2. ***Improve understanding of reproductive biology for the purpose of new pharmaceutical development for MPT R&D*** - Common ground: the opportunity of male contraceptives as MPTs Vahdat & Nickels 3. ***Expand understanding of socio-behavioral research considerations, particularly among groups who have traditionally been underrepresented in MPT research*** - Biomedical, socio-behavioral, and implementation science gaps in multipurpose prevention technology research Cummins et al. - Program impact and potential pitfalls of multi-purpose technologies (MPTs) for HIV prevention and contraception Latka et al. - How might we motivate uptake of the Dual Prevention Pill? Findings from human-centered design research with potential end users, male partners, and healthcare providers Nyagah et al. - End-user research into understanding perceptions of and reactions to a Microarray Patch (MAP) for contraception among women in Ghana, Kenya and Uganda El-Shah et al. 4. ***Expand understanding of market considerations to help ensure successful commercialization and uptake of MPTs*** - Estimating the costs and perceived benefits of oral preexposure prophylaxis (PrEP) delivery in ten counties in Kenya: a costing and a contingent valuation study Forsythe et al. - The role of economic evaluations in advancing HIV multipurpose prevention technologies in early-stage development Chapman et al. 5. ***Enhance understanding of innovative approaches for MPT clinical trials that address regulatory and ethical challenges of testing multiple indications in the same trial*** - Efficient regulatory approval of two novel HIV prevention interventions in a resource-limited setting: experiences from Zimbabwe Murombedzi et al.

While the action areas reflect a focus beyond the scientific challenges inherent in product development, research and development continues to be a significant focus (Action Area #1). According to a World Economic Forum analysis, at the current rate of investment, it will take 132 years to close the global gender gap between men and women—due in significant part to high rates of preventable morbidity during pregnancy and maternal mortality (6). This gender gap is exacerbated by underinvestment in health conditions that primarily impact women's health. A recent analysis of NIH funded work found that nearly 75 percent of research on conditions that disproportionately affected one gender vs. another found either underfunding of research for conditions that are more prevalent in women or overfunding of diseases that affect more men (7). While the NIH is a significant funder and supporter of MPTs, only 3% of the 2019–2020 NIH budget was devoted to female contraceptives and non-HIV STIs (8). Similarly, a 2020 analysis of biopharmaceutical investment found that only 5% of industry investment was in women's health with only 1% of global biopharmaceutical investments dedicated to non-cancer health conditions in women (9).

This lack of robust and diverse investment in women's and reproductive health is a broad challenge impacting MPT product development. Developing a safe, effective product is but the first step and the context in which researchers are working is challenging. In this special issue, Dohadwala et al. present advances in the use of monoclonal antibodies (mAbs) for the prevention of infectious diseases and highlight the fact that the MPT field pioneered the use of topical mAbs. However, they also note the high cost of development and manufacturing will be a significant constraint. Complementing this paper is the work by Cameron et al*.*, examining equitable access to MPTs, and a second paper by authors Vahdat and Nickels from the Male Contraceptive Initiative. The limited funding for contraceptive and MPT research has been heavily focused on female end-users and Vahdat and Nickels outline steps and challenges needed to expand the field to include male end-users, including potential areas of synergy where working collaboratively could yield multiplicative advances in both fields.

One of the hurdles constraining the rapid development of MPTs is the traditional focus on lower- and middle-income countries (LMICs) where industry investment has been limited. As noted by Cameron et al*.*, it takes an average of eight to ten years after HIV medicines receive approval in the United States to reach LMICs. This delay highlights the critical need to engage stakeholders across the research, manufacturing, distribution, and procurement spectrum from the beginning, a strategy that they refer to as end-to-end approach predict, prevent, and remove potential roadblocks to product development and access.

Having a better understanding of market considerations to ensure successful commercialization and uptake of MPTs is one of the action areas that builds on the end-to-end approach. In this issue, three articles examine market considerations. Forsythe et al*.* discuss willingness to pay for oral pre-exposure prophylaxis (PrEP)—a key component of many MPTs under development. They found low willingness to pay for oral PrEP among adolescent girls and young women, a key population for MPTs, suggesting that it may be difficult to use traditional market driven approaches for coverage and distribution of MPTs. Chapman et al*.* underscore the importance of incorporating economic evaluations to inform early-stage development and to mitigate potential market failures. Kutywayo et al*.* highlight the fact that healthcare providers serve as critical gatekeepers and can either stimulate or diminish demand. All three papers help inform our understanding of the challenges the field will need to confront in order to support a robust market for MPTs.

Four additional papers examine the importance of socio-behavioral research in understanding the potential for product uptake and use. Having a clear understanding of end-user needs and preferences is critical for developing a successful market strategy. The aforementioned disparities in research and funding for women's and reproductive health have been driven in part by regulatory policies that previously excluded women of childbearing age in clinical trials. In 1977, the U.S. Federal Food and Drug Administration (FDA) banned women of “childbearing potential” from participating in clinical trials (10). While this policy was formally rescinded in 1993 and the number of women participating in clinical trials has increased, infectious disease research continues to have one of the lowest relative ratio of female to male enrollment (−18.68% relative difference) (11). These historic exclusions have driven our limited understanding of the social-behavioral considerations around women's a health, and knowledge gaps in reproductive biology and differences in male and female pharmacokinetics and pharmacodynamics.

In their commentary, Cummins et al*.* provide a high-level summary of environmental and structural barriers to MPT uptake, including the need to better understand socio-behavioral considerations, adherence, and gaps in implementation science that can either drive or impede effectiveness of MPTs Cummins et al. Responding to this, Latka et al*.*, provide a framework for understanding the interplay between user characteristics, method efficacy, use effectiveness and the importance of developing a diverse array of products that can meet changing values, preferences, and needs across a person's life course. In line with this framework and referencing back to the end-to-end research approach characterized by Cameron et al*.*, Nyagah et al*.* present their findings from a human-centered design approach and reinforce the importance of treating end-users as a diverse and heterogenous collection of individuals with sometimes conflicting and competing identities and values. Putting these theories to practice, El-Sahn et al*.* conducted in-person interviews to provide actionable feedback from potential end-users to product developers.

The MPT field faces a challenging regulatory pathway. MPT candidates include diverse delivery platforms and drug combinations representing both approved and new drugs. MPTs may need to meet US federal requirements for safety and efficacy testing of combination products although some product developers have pursued regulatory approval in only ex-US settings. While these alternative pathways may speed approval, the multiplicity of regulatory agencies makes it challenging to get approval for clinical testing and trial design to provide safety data on multiple indications. Individual indications of an MPT must also meet accepted efficacy standards for each of its indications. In our special issue, Murombedzi et al. detail strategies to reduce the approval time in Zimbabwe from a median of several years (516–1,673 days) to register a drug to 133 days for registration of the dapivirine vaginal ring and 159 days for the long-acting cabotegravir (CAB-LA) by leveraging innovative regulatory pathways and capacity strengthening. Although the MPT regulatory and approval pathway is inherently more complicated than single drug examples we can use these lessons and begin to look for efficiencies in the regulatory pathway for MPTs.

The MPT field faces a myriad challenges, but these products offer the possibility of revolutionizing women's health by offering prevention for multiple indications in single products- a feature most women favor (12–14). While women represent more than half of the world's population, many women across the world face tenuous access to healthcare (15–17). Increasingly scientists, governments, and multilateral organizations recognize the positive impact that reproductive health and family planning have on entire communities and even larger macro trends such as climate change (18–21). While still modest compared to what is needed, investment and attention to and investment in women's reproductive health is increasing (22). To realize the full potential of MPTs, we must strategically work to move promising products from the laboratory to end-users, improving the diverse health needs and wants of women globally.

## References

[1] Yegros-Yegros A van de Klippe W Abad-Garcia M Rafols I. Exploring why global health needs are unmet by research efforts: the potential influences of geography, industry and publication incentives. Health Res Policy Sys. (2020) 18(47). 10.1186/s12961-020-00560-6 PMC7227286 32414373

[2] Stone A. Advancing Prevention Technologies for Sexual and Reproductive Health: Report of a Symposiumn, Berekely, CA. (2009).

[3] Wellcome Trust and the Initiative for Multipurpose Prevention Technologies. Global forum on multipurpose prevention technologies for reproductive health. Advancing the MPT Agenda; London, UK (2012).

[4] Ehrlichman D Sawyer D. Learn before you leap: the catalytic power of a learning network. Stanf Soc Innov Rev. (2018). 10.48558/813X-NK18

[5] Holt BY Turpin JA Romano J. Multipurpose prevention technologies: opportunities and challenges to ensure advancement of the most promising MPTs. Frontiers in Reproductive Health. (2021) 3. 10.3389/frph.2021.704841 PMC9580637 36304018

[6] World Economic Forum. Women’s Health: Is this the World’s Best – and Most Under-Financed – Investment? (2023). Available online at: https://www.weforum.org/agenda/2023/01/is-this-the-world-s-best-and-most-under-financed-investment-davos23/ (Accessed March 2024).

[7] Marin AA. Gender disparity in the funding of diseases by the U.S. National institutes of health. J Womens Health. (2021) 30(7):956–63. 10.1089/jwh.2020.8682 PMC8290307 33232627

[8] Office of Research on Women’s Health. Report of the Advisory Committee on Research on Women’s Health: Fiscal Years 2019–2020. Bethesda, MD: NIH Publication No. 21-OD-7995, National Institutes of Health, Office of Research on Women’s Health and NIH Support for Research on Women’s Health (2021).

[9] McKinsey & Company. Unlocking opportunities in women’s healthcare. (2022). Available online at: https://www.mckinsey.com/industries/healthcare/our-insights/unlocking-opportunities-in-womens-healthcare (Accessed March 19, 2024).

[10] Schiebinger L. Women’s health and clinical trials. J Clin Invest. (2003) 112(7):973–7. 10.1172/JCI19993 14523031 PMC198535

[11] Steinberg JR Turner BE Weeks BT Magnani CJ Wong BO Rodriguez F Analysis of female enrollment and participant sex by burden of disease in US clinical trials between 2000 and 2020. JAMA Netw Open. (2021) 4:6. 10.1001/jamanetworkopen.2021.13749 PMC8214160 34143192

[12] Minnis AM Krogstad E Shapely-Quinn MK Agot K Ahmed K Wagner LD Giving voice to the end-user: input on multipurpose prevention technologies from the perspectives of young women in Kenya and South Africa. Sex Reprod Health Matters. (2021) 29(1). 10.1080/26410397.2021.1927477 34224341 PMC8259853

[13] Bhushan NL Ridgeway K Leucke EH Palanee-Phillips T Montgomery ET Minnis AM. Synthesis of end-user research to inform future multipurpose prevention technologies in sub-saharan Africa: a scoping review. Front Reprod Health. (2023) 5. 10.3389/frph.2023.1156864 37325244 PMC10264572

[14] CAMI Health. (2023). Word on the Street: Women Share Their Reproductive Health Stories. The IMPT. (2023). Available online at: https://theimpt.org/word-on-the-street-story-map/ (Accessed March 22, 2024)

[15] RAND. The Unmet Need for Contraception in Developing Countries. (1998). Available online at: https://www.rand.org/pubs/research_briefs/RB5024.html (Accessed March 19, 2024)

[16] Kreitzer RJ Smith CW Kane KA Saunders TM. Affordable but inaccessible? Contraception deserts in the US States. J Health Polit Policy Law. (2021) 46(2):277–304. 10.1215/03616878-8802186 32955562

[17] Haakenstad A Angelino O Irvine CM Bhutta ZA Bienhoff K Bintz C. Measuring contraceptive method mix, prevalence, and demand satisfied by age and marital status in 204 countries and territories, 1970–2019: a systematic analysis for the global burden of disease study 2019. Lancet. (2022) 400(10348):295–327. 10.1016/S0140-6736(22)00936-9 35871816 PMC9304984

[18] Bongaarts J Sitruk-Ware R. Climate change concerns have largely ignored role of access to effective contraception. BMJ Sex Reprod Health. (2019) 45:233-235. 10.1136/bmjsrh-2019-200399 31615904

[19] Proctor RN Schiebinger L. How Preventing Unwanted Pregnancies Can Help on Climate. Yale School of the Environment. (2022). Available online at: https://e360.yale.edu/features/unwanted-pregnancy-contraception-abortion-climate-change (Accessed March 19, 2024).

[20] Starbird E Norton M Marcus R. Investing in family planning: key to achieving the sustainable development goals. Glob Health Sci Pract. (2016) 4(2):191–210. 10.9745/GHSP-D-15-00374 27353614 PMC4982245

[21] United States Agency for International Development. Helping People and the Planet Flourish Through Family Planning. Available online at: https://www.usaid.gov/global-health/health-areas/family-planning/resources/helping-people-planet-flourish-family-planning (Accessed March 19, 2024).

[22] Schroeder M Anderson M. Where investment in women’s health will be focused in 2023. Healthcare Brew. (2024). Available online at: https://www.healthcare-brew.com/stories/2024/02/27/vc-funding-in-women-s-health-on-the-uprise-in-2023 (Accessed March 19, 2024).

